# IFN Regulatory Factors 4 and 8 Expression in the NOD Mouse

**DOI:** 10.1155/2011/374859

**Published:** 2011-05-15

**Authors:** Gilles Besin, Simon Gaudreau, Émilie Dumont-Blanchette, Michael Ménard, Chantal Guindi, Gilles Dupuis, Abdelaziz Amrani

**Affiliations:** ^1^Department of Pediatric, Immunology Division, Faculty of Medicine and Health Sciences, University of Sherbrooke, 3001 12th Avenue North, Sherbrooke, QC, Canada J1H 5N4; ^2^Institut Armand-Frappier, Institut National de la Recherche Scientifique, 531 boulevard des Prairies, Laval, QC, Canada H7V 1B7

## Abstract

Dendritic cells (DCs) contribute to islet inflammation and its progression to diabetes in NOD mouse model and human. DCs play a crucial role in the presentation of autoantigen and activation of diabetogenic T cells, and IRF4 and IRF8 are crucial genes involved in the development of DCs. We have therefore investigated the expression of these genes in splenic DCs during diabetes progression in NOD mice. We found that IRF4 expression was upregulated in splenocytes and in splenic CD11c^+^ DCs of NOD mice as compared to BALB/c mice. In contrast, IRF8 gene expression was higher in splenocytes of NOD mice whereas its expression was similar in splenic CD11c^+^ DCs of NOD and BALB/c mice. Importantly, levels of IRF4 and IRF8 expression were lower in tolerogenic bone marrow derived DCs (BMDCs) generated with GM-CSF as compared to immunogenic BMDCs generated with GM-CSF and IL-4. Analysis of splenic DCs subsets indicated that high expression of IRF4 was associated with increased levels of CD4^+^CD8*α* ^−^IRF4^+^CD11c^+^ DCs but not CD4^−^CD8*α* ^+^IRF8^+^CD11c^+^ DCs in NOD mice. Our results showed that IRF4 expression was up-regulated in NOD mice and correlated with the increased levels of CD4^+^CD8*α* ^−^ DCs, suggesting that IRF4 may be involved in abnormal DC functions in type 1 diabetes in NOD mice.

## 1. Introduction

Dendritic cells (DCs) are professional antigen presenting cells (APCs) that play a key role in the induction of innate and adaptive immunity [1]. DCs recognize various pathogens and their components through pattern-recognition receptors, such as TLRs. Captured pathogens are processed and presented as antigenic peptides associated with MHC molecules to T cells. Consequently, DCs/T cell interaction leads to Th1 or Th2 responses and also to tolerance by inducing Treg differentiation. The great diversity of immune responses following DCs antigen presentation is attributed to the presence of multiple DCs subsets [2, 3]. At least six DCs subsets have been identified in the mouse spleen including conventional CD11c^high^ DCs and CD11c^int^B220^+^CD11b^−^ plasmacytoid DCs (pDCs) [4]. Conventional CD11c^high^ are divided into three distinct subtypes, CD4^+^CD*8α* ^−^CD11b^high^, CD4^−^CD8*α* ^+^CD11b^low^, and CD4^−^CD8*α* ^−^CD11b^high^ [4–7]. Conventional lymphoid CD8*α* ^+^ and myeloid CD8*α* ^−^DCs are the major producers of IL-12 whereas pDCs are the producers of type I IFN [5–7]. Despite much progress in understanding the biology of DCs, molecular events that specify DC development and functions are not fully understood. Interferon regulatory factors 4 and 8 (IRF4 and IRF8), two members of the IRF transcription factor family involved in the regulation of both innate and adaptive immunity [8], have been shown to contribute to DCs development and function [9–13]. Notably, IRF4 has been shown to be required for the development of CD11b^high^CD8*α* ^−^ DCs subset [13], whereas IRF-8 is required for differentiation of CD11c^+^CD11b^low^CD8*α* ^+^ DCs and pDCs subsets [14]. Indeed, mice lacking the IRF4 gene have selective defect in splenic CD11b^high^CD8*α* ^−^ conventional DCs [13] whereas IRF8^−/−^ mice have a defect in both lymphoid and plasmacytoid DC subsets [14]. Furthermore, CD8*α* ^+^ DCs and pDCs express high levels of IRF8, but low level of IRF4. Conversely, IRF4 expression is high in CD4^+^ DCs and CD4^−^CD8*α* ^−^CD11b^high^ DCs whereas IRF8 expression is low. 

The Nonobese Diabetic (NOD) mouse spontaneously develops diabetes between 15 to 20 weeks of age. The onset of the disease is preceded by a period of insulitis during which the islets of Langerhans are infiltrated by autoreactive T cells and APCs [15]. Interestingly, DCs are the first cells to infiltrate the islets [16], preceding T cell infiltration [17]. Infiltrating DCs produce cytokines such as IL-12 [17], suggesting their implication in the early pathogenesis of diabetes. Furthermore, the administration of IL-12 to NOD mice results in enhanced DCs accumulation in pancreatic islets and accelerated diabetes onset [18]. Although the precise mechanism leading to the breakdown of tolerance to islets antigen is not fully understood, defects in function and maturation of DCs in NOD mice have been suggested [19]. Several studies have shown several abnormalities in BM-derived and splenic DCs of NOD mice as compared to DCs of diabetes-resistant mice [20, 21]. For example, it has been reported that the number of splenic CD8*α* ^+^ DCs and CD8*α* ^−^ DCs in NOD mice is low as compared to those of the diabetes-resistant B10.BR and C57BL6J mice [22]. This decreased population of DCs could result in their reduced ability to take up and to clear dead cells [14] and to maintain self-tolerance [23]. 

In the present study, we have investigated the expression of IRF4 and IRF8 genes in splenocytes and DCs of diabetes-prone NOD mice and compared the results to diabetes-resistant NOR and BALB/c mice. We found an upregulated expression of IRF4 in the total splenic cell population of NOD mice as compared to BALB/c mice. Enhanced IRF4 expression was found in CD11c^+^ splenic DCs of NOD as compared to BALB/c mice. However, IRF4 and IRF8 expression was only increased in BMDCs generated with a combination of IL-4 and GM-CSF whereas their expression was low in BMDCs generated with GM-CSF alone. 

## 2. Materials and Methods

### 2.1. Mice

Male and female NOD, NOR, C57BL/6J, CD1, and BALB/c mice were purchased from the Jackson Laboratories (Bar Harbor, ME). All mice were housed and bred in-house under specific pathogen-free conditions and were used according to guidelines of the Institutional Animal Care Committee of the University of Sherbrooke.

### 2.2. Cell Lines and Antibodies

Anti-CD8*α*-PE (clone 53-6.7), anti-CD4-FITC/biotin/APC (clone GK1.5), anti-CD11c-FITC/biotin (clone HL3) antibodies, and streptavidin-PerCP (used for all biotin conjugated antibodies) were from Becton-Dickinson (San Jose, CA). The anti-IRF4-biotin (clone M17) and anti-IRF8-biotin (clone C19) were from Santa Cruz Biotechnology (Santa Cruz, CA). The antigoat-HRP antibody was from R and D Systems (Minneapolis, MN).

### 2.3. Splenic DCs Isolation

DCs purification was performed using antibody-coated magnetic beads from Miltenyi Biotec (Bergisch Gladbach, Germany) as before [24]. Briefly, spleens were digested with collagenase D (2 or 3 organs), stained with anti-CD11c-coated beads and sorted by MACS (Miltenyi Biotec). Purity of CD11c^+^ DCs was >85% as determined by FACS.

### 2.4. Generation of Bone Marrow-Derived DCs

BMDCs were generated with GM-CSF (5 ng/mL) alone or in combination with IL-4 (4.5 ng/mL) (Cederlane, Burlington, ON) as described [25, 26]. On Day 7, DCs were collected for further analysis.

### 2.5. Real-Time PCR

Total RNA was extracted from DCs using the TRIzol reagent (Invitrogen, Carlsbad, CA). Two micrograms of RNA were reversely transcribed to cDNA using Superscript II (Invitrogen Burlington, ON). Quantitative PCR reactions were performed using Rotor-Gene 3000 (Corbett Life Science, Mortlake, New South Wales, Australia) with a 25 *μ*L mixture composed of 2 *μ*L of cDNA template, 2 *μ*L PCR buffer, 2 *μ*L of dNTP (10 mmol/L), 0.3 *μ*L of each primer (20 pmol/mL), 0.5 *μ*L SYBR green Quantitect SYBR Green qPCR kit (Qiagen), and 0.1 *μ*L of Taq polymerase. The reactions were carried out with an initial denaturation at 95°C for 5 min, followed by 40 cycles of 30 s at 95°C, 30 s at 58°C, and 30 s at 72°C. Amplification plots were generated using the Rotor-Gene Amplification software v6.0 (Corbett Research). HPRT was used as a reference to obtain the relative fold change for target samples using the comparative C_T_ method. The primers used were 5′AATGGGAAACTCCGACAGTG3′ (IRF4 sense), 5′TAGGAGGATCTGGCTTGTCG3′ (IRF4 antisense), 5′GATCGAACAGATCGACAGCA3′ (IRF8 sense), 5′AGAGCACAGCGTAACCTCGT3′ (IRF8 antisense), 5′GTTGGATACAGGCCAGACTTTGTTG3′ (HPRT sense), and 5′GATTCAACTTGCTCTCATCTTAGGC3′ (HPRT antisense).

### 2.6. Western Blots

BM-derived and splenic DCs were harvested, washed in cold PBS, and resuspended in lysis buffer containing Tris 50 mM, NaCl 0.15 M, DTT 1 mM, Triton X-100 1% (v/v), and a cocktail of protease and phosphatase inhibitors. Cell lysates were fractionated on 10% SDS-PAGE gels, transferred to a nitrocellulose membrane (Hybond-ECL Amersham Biosciences, Baie d'Urfé, QC) and incubated overnight with primary antibodies, followed by the appropriate secondary antibodies and revealed by enhanced chemiluminescence (GE Health Care, Baie d'Urfé, QC). Quantification of Western band intensities was performed by densitometry analysis of X-ray films using the NIH Image software (http://rsb.info.nih.gov/nih-image/). 

### 2.7. Flow Cytometry

Cells were washed once with PBS supplemented with 2.5% bovine serum albumin (PBS-BSA) and incubated with the indicated mAbs for 20 min at 4°C. In the case of IRF4 and IRF8 intracellular staining, the cells were fixed with 4% paraformaldehyde for 1 h at 4°C, permeabilized with FACS buffer containing 0.1% saponin and stained with anti-IRF4 or IRF8 Abs or antirat IgG2a for 30 min at 4°C. The cells were then washed twice with PBS-BSA and analyzed by FACS using the CellQuest software (BD Biosciences) or the FCS express V3 software (De Novo Software, Los Angeles, CA). 

### 2.8. Statistical Analysis

Two groups were compared using two-tailed unpaired Student's *t* test. When more that two groups were compared, one-way ANOVA Dunnett's test for multiple comparisons was used. Differences were considered to be statistically significant when a *P* < .05. Data reported here are representative of 2-3 independent experiments. Histograms and bar graph results are shown as the mean ± SEM. 

## 3. Results

### 3.1. IRF4 But Not IRF8 Gene Expression Is Increased in Splenocytes of NOD Mice

**Figure 1:**
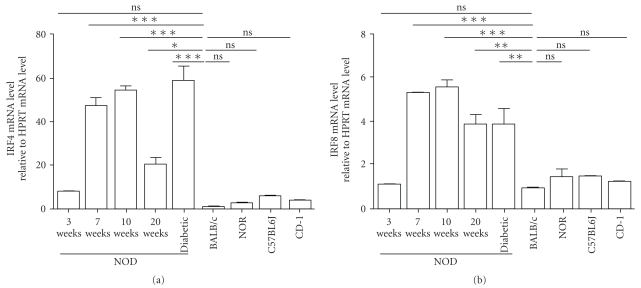
Age-related IRF4 and IRF8 mRNA expression levels in splenocytes of diabetes-prone and diabetes-resistant mice. Total splenic RNA was obtained from NOD, NOR, C57BL/6J, CD-1, and BALB/c mice, purified and gene expression profile of IRF4 (a) and IRF8 (b) were analyzed by real time RT-PCR. Each RNA sample was analyzed in triplicates and normalized to HPRT expression (ΔCt). Each NOD sample was normalized to a BALB/c sample (ΔΔCt = ΔCt sample − ΔCt BALB/c) and, the expression level was calculated as follows: 2^−ΔΔCt^. Data are shown as the average ± SD of 4 independent experiments (*P < .05, **P < .01 and ***P < .001).

In a first series of experiments, we used quantitative PCR (qPCR) to assess gene expression of IRF4 and IRF8 in splenocytes isolated from prediabetic and diabetic NOD mice as well as control diabetes-resistant congenic NOR and BALB/c mice. We found that the levels of mRNA expression of IRF4 were slightly but not significantly higher (*P* > .05) in splenocytes of 3-week old NOD (7.7 ± 0.8) mice as compared to splenocytes of diabetes-resistant BALB/c (1.04 ± 0.1), NOR (2.6 ± 0.6), C57BL/6 (5.8 ± 0.9), or CD1 (3.7 ± 0.8) mice (Figure 1(a)). The levels of IRF4 mRNA expression were significantly (*P* < .001) increased in the splenocytes of 7-, 10-, and 20-week old diabetic NOD mice (54.8 ± 1.9 and 58.9 ± 16.1, resp.) as compared with 3 weeks old NOD mice and diabetes-resistant mice (Figure 1(a)). However, the expression of IRF4 remained unchanged in the splenocytes of 3-, 6-, 10-, or 20-week old NOR and BALB/c mice (data not shown). Interestingly, IRF4 gene expression in splenocytes of nondiabetic 20-week old NOD mice was significantly lower (*P* < .05) than in 20-week old diabetic NOD mice (20 ± 5.3 compared to 53.9 ± 16.1) (Figure 1(a)).

Analysis of IRF8 gene expression revealed similar levels of IRF8 mRNA in splenocytes of BALB/c and 3-week old NOD mice (0.9 ± 0.1 as opposed to 1.1 ± 0.1) (Figure 1(b)). In addition, IRF8 gene expression was significantly (*P* < .05) increased in the splenocytes of 7-, 10-, and 20-week old NOD mice (Figure 1(b)). In contrast, the levels of IRF8 gene expression were similar in the splenocytes of 20-week old nondiabetic and diabetic NOD mice (3.8 ± 0.6 as opposed to 3.8 ± 1.1) (Figure 1(b)).

**Figure 2:**
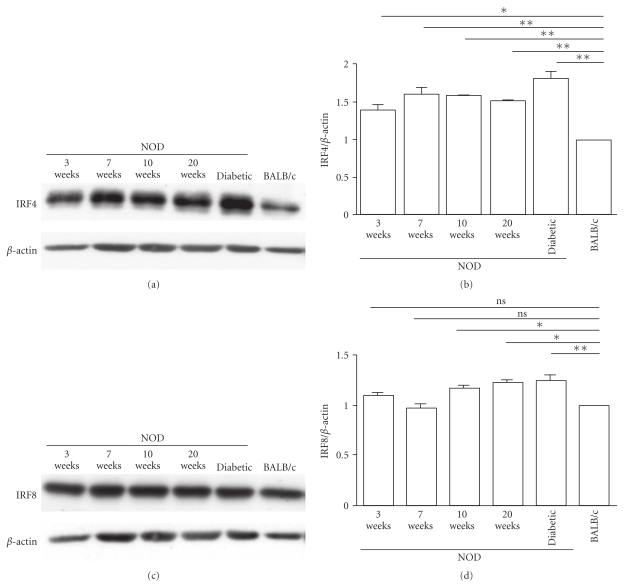
Age-related IRF4 and IRF8 expression levels in splenocytes of diabetes-prone NOD mice and diabetes-resistant BALB/c mice. Proteins were extracted from NOD or BALB/c mouse splenocytes and analyzed by Western blotting for IRF4 (a) or IRF8 (c) expression. The relative intensities of the bands were determined using the NIH Image software and normalized to reference actin bands to establish a ratio of (b) IRF4/actin and (d) IRF8/actin. The levels of expression in BALB/c mice were arbitrarily set at a unitary value. Data are representative of 3 independent experiments.

We next determined whether changes observed in the expression of the IRF4 and IRF8 genes were matched by changes at the protein levels. Total proteins extracts were prepared from the splenocytes of NOD and BALB/c mice and the expression of IRF4 and IRF8 was analyzed by Western blots (Figure 2). Results showed that IRF4 expression was significantly higher (*P* < .05) in splenocytes of 3-week old NOD mice than in BALB/c mice (1.38 ± 0.08 as opposed to 1.00 ± 0) (Figures 2(a) and 2(b)). IRF4 expression was slightly but not significantly (*P* > .05) higher in 7-, 10-, and 20-week old nondiabetic and diabetic NOD mice (1.58 ± 0.02, 1.60 ± 0.1, 1.52 ± 0.03, and 1.81 ± 0.10, resp.) as compared to IRF4 expression in 3-week old NOD mice (Figures 2(a) and 2(b)). In contrast, the protein levels of IRF8 in splenocytes of 3- and 7-week-old NOD mice were similar to those of BALB/c mice (1.09 ± 0.1, 0.98 ± 0.10 as compared to 1.00 ± 0, *P* > .05). Protein levels of IRF8 were significantly higher (*P* < .05) in 10- and 20-week-old non diabetic and diabetic NOD mice (1.16 ± 0.1, 1.22 ± 0.03, and 1.25 ± 0.10, resp.) as compared to BALB/c (1.00 ± 0) mice (Figures 2(c) and 2(d)). Together, these results suggested that gene and protein expression levels of IRF4 were upregulated in the spleen of diabetes-prone NOD mice whereas levels of IRF8 remained unchanged at 3 and 7 weeks of age, and were slightly increased in 10 and 20 weeks old mice when compared to BALB/c mice.

### 3.2. Increased Expression of IRF4 Correlated with Augmented CD4^+^CD8*α* ^−^CD11c^+^ Splenic DCs in NOD Mice

**Figure 3:**
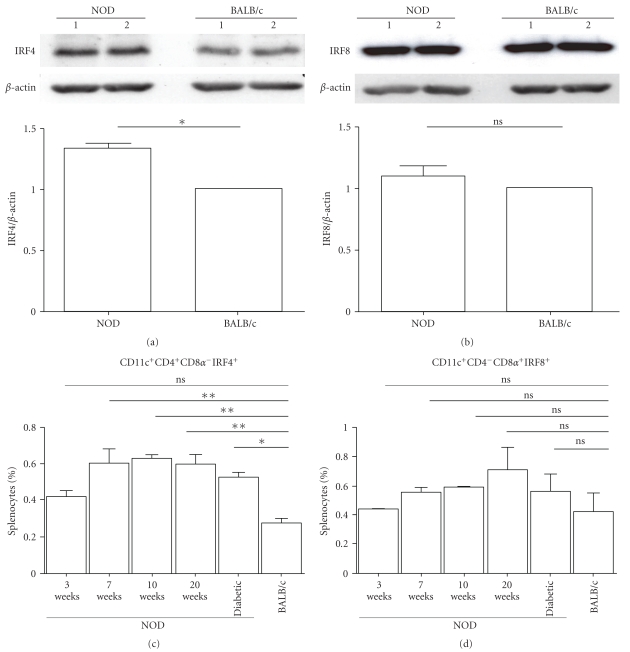
Expression of IRF4 in splenic DCs of diabetes-prone NOD mice and diabetes-resistant BALB/c mice. Proteins were extracted from CD11c^+^-purified splenic DCs from NOD and BALB/c mice and analyzed by Western blotting for (a) IRF4 or (b) IRF8 expression. The relative intensities of the bands were assessed using the NIH Image software and normalized to reference actin bands to establish a ratio of IRF4/actin (a), lower panel) and (b), lower panel) IRF8/actin. The expression levels observed in BALB/c were arbitrarily set as a unitary value. Data are representative of 2 independent experiments (Exp1 and Exp2). (c) and (d) splenocytes from NOD and BALB/c mice were stained for the CD11c, CD4, and CD8*α* surface markers in combination with intracellular staining with an anti-IRF8 or anti-IRF4 mAb and analyzed by flow cytometry. Data represent the average percentage of CD11c^+^CD4^+^CD8*α* ^−^IRF4^+^-positive (c) and CD11c^+^CD4^−^CD8*α* ^+^IRF8^+^-positive (d) cells of two independent experiments. Error bars correspond to the averages ±S.D (*P < .05, **P < .01 and ***P < .001).

To determine whether differential expression of IRF4 and IRF8 in splenocytes of NOD mice resided in splenic DCs, we examined the levels of protein expression of IRF4 and IRF8 by Western blots in CD11c^+^-purified splenic DCs of 8-week-old NOD and BALB/c mice. Results showed (Figure 3(a)) that the expression of IRF4 was higher in splenic DCs of NOD mice as compared to BALB/c mice (*P* < .05). In contrast, IRF8 expression was similar in splenic DCs of both strains of mice (Figure 3(b)). Since IRF4 and IRF8 have been shown to be essential for the development of CD4^+^CD8*α* ^−^CD11c^+^ and CD4^−^CD8*α* ^+^CD11c^+^ subsets, respectively [9], we determined whether the changes observed in IRF4 and IRF8 expression in splenic DCs of NOD and BALB/c mice affected the proportions of these two DCs subsets. Results of FACS analysis (Figure 3(c)) showed no difference in the percentage of CD4^+^CD8*α* ^−^IRF4^+^CD11c^+^ DCs subset in splenocytes of 3-week-old NOD and BALB/c mice (0.42% ± 0.03 as opposed to 0.27% ± 0.03, *P* > .05). The percentages of splenic CD4^+^CD8*α* ^−^IRF4^+^CD11c^+^ DCs subset were significantly (*P* < .05) increased in 7-, 10-, and 20-week-old NOD mice (0.60% ± 0.030 at 7 weeks, 0.63% ± 0.03 at 10 weeks, and 0.60% ± 0.07 at 20 weeks) as compared to 3 weeks old (NOD 0.42% ± 0.03) and BALB/c (0.27% ± 0.0) mice. In addition, there were no significant difference in splenic CD4^+^CD8*α* ^−^IRF4^+^CD11c^+^ DCs subset in 7-, 10-, and 20-week-old nondiabetic and diabetic NOD mice. In contrast, an absence of differences was noted in the percentage of CD4^−^CD8*α* ^+^IRF8^+^CD11c^+^ DCs subset in the splenocytes of BALB/c mice and diabetic or nondiabetic NOD mice (Figure 3(d)). Together, these data suggested that high expression of IRF4 in NOD mice was associated with enhanced splenic CD4^+^CD8*α* ^−^IRF4^+^CD11c^+^ DCs subset but not with splenic CD4^−^CD8*α* ^+^IRF8^+^CD11c^+^ DCs subset. 

### 3.3. Increased Expression of IRF8 and IRF4 in BMDCs of NOD Mice

**Figure 4:**
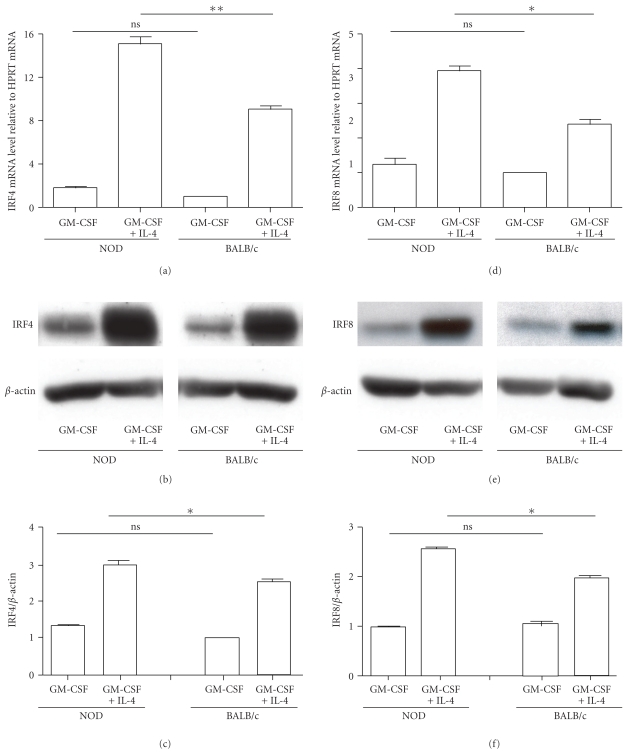
Reduced expression of IRF4 and IRF8 in tolerogenic BMDCs derived from NOD mice. Total RNA was extracted from BM-DCs generated with GM-CSF or a combination of GM-CSF and IL-4, purified and gene expression profiles of IRF4 (a) and IRF8 (d) were analyzed by RT-PCR. Each sample was analyzed in triplicates and normalized to HPRT expression (ΔCt). Each NOD sample was normalized to BALB/c sample (ΔΔCt = ΔCt sample − ΔCt BALB/c), and the expression levels were calculated as follows: 2^−ΔΔCt^. Data are shown as the average ±SD of 3-4 independent experiments (*P < .05 and, **P < .01). (b) and (e) total proteins were extracted from BM-DCs generated with GM-CSF or a combination of GM-CSF and IL-4 and analyzed by Western blotting for IRF4 (b) or IRF8 (f) expression. The relative intensities of bands were assessed using the NIH Image software and normalized to reference actin bands to establish a ratio of (c) IRF4/actin and (f) IRF8/actin. The expression levels observed in BALB/c mice were arbitrarily set as a unitary value. A representative of 2 independent experiments is shown. Error bars correspond to S.D.

Defects in phenotype and tolerogenic function of BMDCs of NOD mice have also been associated with diabetes [20, 21]. We have reported that treatment of NOD mice with GM-CSF restored the tolerogenic function of myeloid DCs [24]. In addition, BMDCs generated with low dose of GM-CSF possessed tolerogenic functions when compared to immunogenic BMDCs generated with GM-CSF and IL-4 [27] (Guindi et al., unpublished data). Therefore, we determined the expression IRF4 and IRF8 in BMDCs of NOD mice generated with GM-CSF or with a combination of GM-CSF and IL-4. BMDCs generated from BALB/c mice were used as controls. Under both sets of experimental conditions, more than 95% of BMDCs were CD8*α* ^−^CD11c^+^ cells. Results showed that BMDCs generated with GM-CSF from both strains of mice expressed similar levels of IRF4 as determined by qPCR (Figure 4(a)) and by Western blot (Figures 4(b) and 4(c)). IRF4 expression was increased in BMDCs generated with a combination of GM-CSF and IL-4 and was significantly higher (*P* < .05) in NOD as compared to BALB/c mice (Figures 4(a), 4(b) and 4(c)). The expression of IRF8 was similar in BMDCs of both strain of mice generated with GM-CSF (Figures 4(d), 4(e) and 4(f)). However, BMDCs generated with a combination of GM-CSF and IL-4 expressed significantly (*P* < .05) higher levels of IRF8 as compared to BMDCs generated with GM-CSF. Importantly, the levels of expression of IRF8 were higher in the case of immunogenic BMDCs generated with GM-CSF and IL-4 from NOD than in the case of BALB/c mice (Figures 4(d), 4(e) and 4(f)). Together, these results showed that IRF4 and IRF8 were highly expressed in immunogenic BMDCs generated with a combination of GM-CSF and IL-4 in the case of both strains of mice, whereas their expression was significantly lower in the case of tolerogenic BMDCs generated with GM-CSF alone.

## 4. Discussion

In this study, RNA gene expression and protein analysis were used to investigate the expression profile of the two transcription factors IRF4 and IRF8 that are known to play an important role in DCs differentiation [9–13]. We found that the expression of IRF4 was enhanced in splenic DCs and in immunogenic BMDCs but not in tolerogenic BMDCs of NOD mice. The increased IRF4 expression was associated with a greater percentage of CD4^+^CD8*α* ^−^IRF4^+^CD11c^+^ DCs but not CD4^−^CD8*α* ^+^IRF8^−^CD11c^+^ DCs. In contrast, IRF8 expression remained unchanged in splenic DCs of NOD and diabetes-resistant BALB/c mice although it was significantly increased in immunogenic BMDCs but not in tolerogenic BMDCs of NOD mice. 

Development of type 1 diabetes consists in a succession of events during which variation in gene expression plays a critical role in progression from islet inflammation to clinical diabetes [28, 29]. The development of microarray technology has provided a new approach to understand diabetes pathogenesis and to identify genes deregulated in type 1 diabetes including NOD mice [29, 30]. IRF4 is one candidate gene located in the *idd14* susceptibility region suspected to play a role in the development of type 1 diabetes in NOD mice [29]. IRF8, another candidate gene important in regulating Th2 immune response, has been suggested to play an important role in diabetes development [29]. Here, we found high levels of mRNA and protein expression of IRF4 in splenic cells of NOD mice when compared to BABL/c mice. IRF4 expression was particularly found to increase in 7 weeks and older NOD mice in which islet inflammation has already occurred and in diabetic NOD mice. These results suggested that up-regulation of IRF4 expression may play an important role in diabetes development in diabetes-prone NOD mice. Our results could not be explained by triggering immune response toward Th1 response. In this connection, several studies have reported that diabetes in NOD mice is a Th1-mediated disease and that immuno-deviation toward a Th2 response contributes to prevention of diabetes development [31–34]. Of note, IRF4 has been shown to be essential for the Th2 response, and naïve T cells of mice deficient for IRF4 have a compromised production of IL-4 and Th2 cytokines [35]. Alternatively, the increased IRF4 expression during islet inflammation and diabetes development in NOD mice could be also explained by the requirement of IRF4 for production and responsiveness to IL-21 and for stabilization of the Th17 phenotype [36–38] that has been shown to be critical for the development of type 1 diabetes [39]. This observation may also explain the reduced expression of IRF4 in 20-week-old nondiabetic NOD mice as opposed to 20-week-old diabetic NOD mice. In contrast, the increased IRF8 expression in the spleen of diabetes-prone NOD mice may be the result of a high production of IFN*γ* which is known to induce IRF8 expression in macrophages and T cells [40].

IRF4 and IRF8 play a key role in molecular programs regulating DCs development and function [9–13]. Abnormal DCs development and function have been reported to be associated with diabetes in human and in NOD mice [20, 21, 41]. Here, we found that splenic DCs of NOD mice expressed high levels of IRF4 when compared to DCs of nondiabetic NOD mice and BALB/c mice, whereas the expression of IRF8 was similar in splenic DCs of NOD and BALB/c mice. The expression of IRF4 in NOD mice was associated with an increase in the number of the CD4^+^CD8*α* ^−^IRF4^+^CD11c^+^ DCs subset, thereby confirming that IRF4 contributed to the development of CD4^+^CD11c^+^ DCs [9, 42]. Furthermore, our data suggested that increases in IRF4 expression and CD4^+^CD8*α* ^−^CD11c^+^IRF4^+^ DCs population in NOD mice were associated with the abnormal function of DCs in diabetes-prone NOD mice. Several studies have also reported an abnormal function of bone marrow-derived DCs of NOD mice generated with a combination of GM-CSF and IL-4. For instance, it was found that the enhanced capacity to activate autoreactive T cells and the increased production of IL-12p70 contributed to the development of diabetes in NOD mice [43, 44]. We also observed that BMDCs of NOD mice, generated with GM-CSF were less immunogenic than BMDCs generated with a combination of GM-CSF and IL-4 (Guindi et al. submitted). This observation prompted us to compare the expression of IRF4 and IRF8 in BMDCs generated with GM-CSF and with a combination of GM-CSF and IL-4. IRF4 and IRF8 were less expressed in BMDCs generated with GM-CSF whereas they were more expressed in BMDCs generated with GM-CSF and IL-4, suggesting that downregulation of IRF4 and IRF8 may attenuate abnormal immunogenic function of BMDCs in NOD mice. In support of this interpretation, several studies have reported that IRF8 contributes to the production of IL-12p70 by acting as transcriptional activator of the IL-12p35 and IL-12p40 genes [45–48]. Therefore, our findings of high expression of IRF8 in DCs of NOD mice may explain their abnormal high production of IL-12p70. Our data also suggested that increased levels of IRF4 in DCs contribute to their abnormal function in NOD mice. In support of this interpretation, the transcription of IRF4 has been shown to be regulated by NF-*κ*B elements located in the IRF4 promoter region that bind Rel/NF-*κ*B complexes [49]. In this context, increased NF-*κ*B activation has been reported to display essential functions of BMDCs for the development of type 1 diabetes [50]. Therefore, enhanced NF-*κ*B activation in DCs of NOD mice leading to their hyperactivation may result from upregulated IRF4 expression.

Our data reported an enhanced involvement of the IRF4 and IRF8 in DCs function in type 1 diabetes-susceptible NOD mice as opposed to control, nondiabetic mice. Further investigation on the role of IRF4 and IRF8 in diabetes development may help to determine whether IRF4 and/or IRF8 could be potential targets for therapeutic interventions in type 1 diabetes.

## Abbreviations

DCs: Dendritic cells
BMDCs: Bone marrow-derived dendritic cells
GM-CSF: Granulocyte macrophage-colony stimulating factor
IRF: Interferon regulating factor.
